# National quality improvement policies and strategies in European healthcare systems

**DOI:** 10.1136/qshc.2008.029355

**Published:** 2009-01-26

**Authors:** E Spencer, K Walshe

**Affiliations:** Herbert Simon Institute for Public Policy and Management, Manchester Business School, Manchester, UK

## Abstract

**Objective:**

This survey provides an overview of the development of policies and strategies for quality improvement in European healthcare systems, by mapping quality improvement policies and strategies, progress in their implementation, and early indications of their impact.

**Study design:**

A survey of quality improvement policies and strategies in healthcare systems of the European Union was conducted in 2005 for the first phase of the Methods of Assessing Response to Quality Improvement Strategies (MARQuIS) project.

**Participants:**

The survey, completed by 68 key experts in quality improvement from 24 European Union member states, represents their views and accounts of quality improvement policies and strategies in their healthcare systems.

**Principal findings:**

There are substantial international and intra-national variations in the development of healthcare quality improvement. Legal requirements for quality improvement strategies are an important driver of progress, along with the activities of national governments and professional associations and societies. Patient and service user organisations appear to have less influence on quality improvement. Wide variation in voluntary and mandatory coverage of quality improvement policies and strategies across sectors can potentially lead to varying levels of progress in implementation. Many healthcare organisations lack basic infrastructure for quality improvement.

**Conclusions:**

Some convergence can be observed in policies on quality improvement in healthcare. Nevertheless, the growth of patient mobility across borders, along with the implications of free market provisions for the organisation and funding of healthcare systems in European Union member states, require policies for cooperation and learning transfer.

The free movement of goods, services, finance and people within the European Union (EU) has profound implications for healthcare financing and healthcare systems in the 27 member states.^1^ The health policies of individual European member states are becoming more interconnected and interdependent, particularly because of the movement of patients and health professionals across national borders. However, each member state still has primary responsibility within its own borders for healthcare funding, and the provision and monitoring of healthcare services, and the EU has quite limited powers to act in this area.^2^ The growing internationalisation of healthcare systems in Europe has an important bearing on the quality and safety of health services.^3^ While many member states have enacted legislation and adopted policies and strategies at a national level, little is known about the relative progress, compatibility, and likely interactions of these national policies and strategies, or their likely implications for EU initiatives.^4^^–^7

We conducted a survey of quality improvement (QI) policies and strategies in European healthcare systems as part of the Methods of Assessing Response to Quality Improvement Strategies (MARQuIS) project.^8^ The study set out to map and describe the policies and strategies in use across the EU, progress made in their implementation, and early indications of their impact. Our aim was to provide a basis for learning and exchange among member states, and to inform future more detailed empirical studies within MARQuIS at an organisational or hospital level.

## METHODS

A literature review was conducted drawing on comparative empirical research, commentaries and theoretical papers, and national or international reports and guidelines on quality in healthcare in Europe, published over a 10-year period (1994–2004).^9^ We searched Medline, ASSIA, HMIC and other databases, using as search terms, various combinations of “quality”, “health services”, “health care” and “Europe”. This literature was used to develop an analytic framework and questionnaire covering six main areas: national environment and context, quality policy goals and values, resources and support, policy implementation, information reporting and evaluation, and impact. For the purposes of this survey “quality improvement policies and strategies” was an umbrella term used to cover a broad range of quality assurance, quality control and quality management approaches to improving healthcare services. We made efforts to define these and other terms used in the questionnaire clearly, but recognised that the meaning and interpretation attached to them by respondents could vary.

We used our own and others’ contacts and networks, the membership of the International Society for Quality in Health Care (ISQua), and web resources to identify key experts in QI in all 25 member states of the EU, including members of international quality societies and well-established contacts in the field. They came from a range of healthcare backgrounds including academia and research, quality societies, policy making, quality consultancies, healthcare management and clinical practice. We approached 174 potential experts in QI by email with information about the survey, of whom 103 agreed to complete the questionnaire; 68 completed questionnaires were returned. The questionnaire survey was conducted in English, which may have limited those experts who were able to take part. Quality experts from 24 member states participated in the survey, and the numbers of respondents were as follows: Austria (3), Belgium (3) Cyprus (1), Czech Republic (2), Denmark (3), Estonia (6), Finland (2), France (3), Germany (2), Greece (1), Hungary (2), Ireland (2), Italy (5), Lithuania (2), Luxembourg (2), Malta (2), the Netherlands (2), Poland (3), Portugal (3), Slovakia (4), Slovenia (3), Spain (4), Sweden (3) and the UK.(5) The survey took place between June and November 2005.

Quantitative data were analysed using SPSS v.10, to produce a range of descriptive and inferential statistics. Qualitative data were subjected to thematic and categorical analysis. For quantitative data, we considered aggregating responses from each country and presenting them at the national level, rather than simply analysing and presenting data for Europe as a whole. However, the numbers of respondents in some individual countries were too small to permit reliable analysis, and we found that in about half of the countries there were important variations in health policies and health systems at a subnational (provincial, state or county) level which made national level analyses potentially misleading. Our full report presents some analyses at both levels.^10^ In this paper, we mainly present the descriptive results from the survey for Europe as a whole, while using some country-level data where it was appropriate to do so, by way of example.

## RESULTS

### National environment and context

The most important drivers for the development of policies and strategies for healthcare quality by governments were reported as being the work of professional organisations—for example: medical and scientific societies; the policies and priorities of current governments; and media coverage of quality issues in healthcare. Less influence was attributed to patient and service user organisations, and to international drivers such as the policies and initiatives of the European Commission, and the activities of ISQua (table 1).

**Table 1:** The level of importance given to different influences on the development of quality improvement policies and strategies (n = 68 respondents)

Influences on the development of quality improvement policies		Very important% (n)	Fairly important% (n)	Not very important% (n)	Not at all important% (n)	Don’t know% (n)
Professional organisations, medical and scientific societies		28 (19)	51 (35)	15 (10)	3 (2)	3 (2)
Policies and priorities of the current government		54 (37)	21 (14)	15 (10)	7 (5)	3 (2)
Media coverage of quality issues or problems in healthcare		25 (17)	44 (30)	25 (17)	3 (2)	3 (2)
National or regional quality task force or working group		35 (24)	32 (22)	19 (13)	10 (7)	3 (2)
Provider organisations, eg, hospitals or primary care providers		22 (15)	44 (30)	24 (16)	7 (5)	3 (2)
Public opinion about the quality of healthcare		19 (13)	44 (30)	25 (17)	7 (5)	4 (3)
Development of quality improvement policies in other EU countries		21 (14)	38 (26)	29 (20)	9 (6)	3 (2)
Patient and service user organisations		7 (5)	43 (29)	40 (27)	7 (5)	3 (2)
Policies and initiatives of the European Commission		10 (7)	22 (15)	53 (36)	12 (8)	3 (2)
Activities of the International Society for Quality in Health Care		4 (3)	25 (17)	44 (30)	24 (16)	3 (2)

Quality improvement policies and strategies in member states can be developed at national governmental level or at a regional level, or from the combined, coordinated or distinctive efforts of national and regional governments. Forty-four per cent of respondents reported the development of quality policies primarily at the national level, 46% at combined national and regional levels, and 10% primarily at the regional level. Table 2 offers some examples of each.

**Table 2:** Variation in the locus for development of quality policies and strategies in government

National	National and regional	Regional
France	Germany	UK
Luxembourg	The Netherlands	Spain
Hungary	Austria	Italy
		Sweden

Fifty-five per cent of respondents reported that there was a great deal or a moderate amount of variation in policies across regions in their country. Some key regional differences were reported in:

- approaches to measuring and evaluating quality;
- priorities across regions and between national and regional levels;
- how national policies were interpreted or implemented at regional level;
- organisation and implementation of QI;
- access to resources to support QI;
- professional motivation, training, and competence.

### Quality policy goals and values

Seventy-eight per cent of respondents reported that in their country there was a statutory legal requirement for healthcare organisations to have QI systems, and such a requirement was in place in at least 18 of the 24 member states covered by our survey. Most commonly (36%), respondents reported that this requirement had been in place for between 5 and 10 years. Hospital services and health services in the public sector appeared to be the main focus of legal requirements in QI, and they less commonly applied to long-term and primary care and to the independent or private healthcare sector.

Respondents were invited to list what they saw as their government’s three most important priorities for QI within their own healthcare systems. The most important priorities identified were:

- development of quality standards and guidelines (18 member states);
- improving patient safety, orientation, and involvement (16 member states);
- improving the assessment and evaluation of QI (9 member states);
- improving information and reporting systems (8 member states);
- achieving better value for money (6 member states).

Two-thirds of respondents (66%) reported that there was a national policy document on QI in healthcare produced by government. As table 3 shows, these national policy documents frequently set out definitions of QI, systems for monitoring and measuring QI, and targets for QI. Rather less frequently they addressed issues such as the provision of training and support for QI, or the resourcing of QI.

**Table 3:** The content of national quality improvement (QI) policy documents (n =  45 respondents who reported having access to a quality improvement policy document)

Topics in a policy document on quality improvement		Topic is included% (n)	Topic is not included% (n)	Don’t know% (n)
Systems for monitoring and measuring progress of QI		82 (37)	13 (6)	4 (2)
Definition of terms, eg, what is meant by QI		78 (35)	18 (8)	4 (2)
Setting national targets for QI		73 (33)	27 (12)	0
Systems for asking patients and the public for their views on quality in healthcare		73 (33)	24 (11)	2 (1)
Setting national standards for quality		71 (32)	27 (12)	2 (1)
Systems for dealing with adverse events, problems and complaints from patients		62 (28)	36 (16)	2 (1)
Systems for professional regulation and monitoring professional performance		56 (25)	42 (19)	2 (1)
Setting standards for professional education or training		53 (24)	38 (17)	9 (4)
Provision of training or support for healthcare organisations on QI		44 (20)	56 (25)	0
Provision of resources for QI		40 (18)	56 (25)	4 (2)

QI, quality improvement.

### Policy implementation

It was noted earlier that respondents reported that most countries had some statutory requirement for healthcare providers to have systems of QI in place, particularly in the public hospital sector. We identified the six most commonly used QI systems in our literature search, and then asked respondents to indicate whether they were mandatory (their use was required) or voluntary (they could be used if the organisation wanted to do so) in hospitals. As table 4 shows, none of these QI systems was widely mandated in the hospital sector, and there was a heavy reliance on voluntary participation and implementation. Patient satisfaction surveys and performance indicator measures were reported as rather more likely to be mandatory, while accreditation and organisational quality or total quality management programmes were much less so. The six most commonly used QI systems that we identified in the literature were also used as a framework in subsequent work packages for the MARQuIS project.^11^

**Table 4:** The mandatory use of quality improvement policies and strategies in hospital services (n = 68 respondents)

Quality improvement policy and strategy in hospitals		Required in hospitals% (n)	Voluntary in hospitals% (n)	Not applicable/Don’t know% (n)
Systems for getting the views of patients, eg, satisfaction surveys, monitoring complaints		50 (34)	43 (29)	7 (5)
Performance indicators or measures		47 (32)	46 (31)	7 (5)
Patient safety systems, eg, incident reporting, risk management		44 (30)	47 (32)	9 (6)
Clinical guidelines, practice guidelines		40 (27)	54 (37)	6 (4)
Accreditation schemes and programmes		27 (18)	54 (37)	19 (13)
Audit, internal assessment of clinical standards		25 (17)	63 (43)	12 (8)
Organisational quality management programmes, eg, total quality management		22 (15)	66 (45)	12 (8)

Respondents were also asked about the extent to which a wide range of measures to support the implementation of QI were present in hospitals in their country. As table 5 shows, they tended to report that longstanding traditional QI systems such as committees for infection control, arrangements for equipment maintenance and laboratory quality control procedures were well established. However, the fundamental components of a QI programme, including an organised system for undertaking QI projects, resources for QI activities, regular QI reviews of departments, and training in QI methods were all reported to be much less commonly found in hospitals.

**Table 5:** The coverage of measures to support the implementation of quality improvement (QI) policies and strategies in the hospital sector (n = 68 respondents)

Measure of support		In all or most hospitals% (n)	In many hospitals% (n)	In some hospitals% (n)	In a few hospitals% (n)	In none% (n)	Don’t know% (n)
Committee for infection control		57 (39)	25 (17)	13 (9)	1 (1)	0	3 (2)
Systems for QI in laboratories		38 (26)	38 (26)	12 (8)	4 (3)	0	7 (5)
Regular maintenance of clinical equipment		40 (27)	32 (22)	9 (6)	10 (7)	1 (1)	7 (5)
Committee for QI		21 (14)	31 (21)	18 (12)	24 (16)	3 (2)	4 (3)
Reporting systems for quality problems such as adverse incidents or events		28 (19)	18 (12)	19 (13)	25 (17)	6 (4)	4 (3)
Staff training in QI		9 (6)	32 (22)	35 (24)	19 (13)	0	4 (3)
Clear responsibilities for clinical performance		24 (16)	16 (11)	16 (11)	25 (17)	12 (8)	7 (5)
Director or leader of QI at a senior level in the organisation		7 (5)	28 (19)	24 (16)	34 (23)	4 (3)	3 (2)
A QI plan for the organisation		7 (5)	24 (16)	31 (21)	29 (20)	2 (1)	7 (5)
Information systems to provide data on quality of care		9 (6)	19 (13)	22 (15)	35 (24)	9 (6)	6 (4)
Regular staff performance reviews		13 (9)	13 (9)	24 (16)	35 (24)	7 (5)	7 (5)
Training for leadership in QI		4 (3)	21 (14)	38 (26)	22 (15)	9 (6)	6 (4)
Regular internal quality reviews of departments or parts of the organisation		6 (4)	18 (12)	37 (25)	28 (19)	7 (5)	4 (3)
An organised programme of QI projects		9 (6)	13 (9)	32 (22)	35 (24)	4 (3)	6 (4)
Systematic follow-up and re-auditing of QI projects		4 (3)	9 (6)	22 (15)	44 (30)	15 (10)	6 (4)
Dedicated finance or budget for QI		4 (3)	6 (4)	19 (13)	49 (33)	15 (10)	7 (5)

QI, quality improvement.

### Progress and impact

Respondents were invited to reflect and report on the three most important achievements in QI in their healthcare system within the past 3 years. The most frequently cited achievements in QI were:

- establishing national accreditation or quality assurance systems (17 member states);
- establishing a national society for quality in healthcare (13 member states);
- extending patient choice, patient rights and patient safety (13 member states);
- improving the training and assessment of professionals (12 member states).

These and the wide range of other achievements reported tended to be concerned with the creation or development of systems, processes and capability in QI, rather than more directly with the outcomes of improvement themselves.

Respondents were also asked to report on the main factors which had acted either as facilitators or barriers to the progress of healthcare QI; the results are summarised in table 6. Respondents saw the key facilitators of progress as professional involvement and training, the existence of a statutory legal requirement for QI, and public demand or expectations, while the main barriers were a lack of funding, poor political leadership and strategic planning, and a lack of incentives or motivations for healthcare organisations and professionals to engage with QI.

**Table 6:** Factors identified by respondents as facilitators or barriers to the progress of healthcare quality improvement (QI)

Facilitators		Barriers
Professional involvement, training, and initiatives (16 member states)		Under-funding (17 member states)
A legal requirement to implement QI (15 member states)		Lack of political leadership and strategic planning (15 member states)
Public demand, expectations and involvement (12 member states)		Lack of incentives, confused incentives, low motivation (12 member states)
Quality improvement projects, eg, accreditation, licensing, awards, quality assurance, circles and forums, quality committees, improvement centres (10 member states)		Cultural barriers, eg, professional or bureaucratic (11 member states)
Political interest (9 member states)		Lack of professional training or education (10 member states)
Harmonisation of policy across the EU, progress in other member states, international guidelines (7 member states)		Under-staffing, time issues, neglect of staff interests (10 member states)
A national strategy for QI (7 member states)		Inadequate management and governance structures (9 member states)
A national society for quality (6 member states)		Lack of clarity in standards, accountability, controls, and priorities (5 member states)
Financial incentives to implement QI (6 member states)		Weak public pressure (5 member states)
Strong leadership (5 member states)		Punitive and negative approaches to monitoring quality or errors (5 member states)
Data on clinical performance (5 member states)		Lack of coordination; networking at organisational, local, and regional levels (5 member states)
Having clear and explicit QI policies (4 member states)		Political change and transition (4 member states)
		Lack of and fear of transparency (4 member states)
		Inadequate or uncoordinated data on quality (4 member states)

## DISCUSSION

**Key messages**

- There are substantial international and intra-national variations in the development of healthcare quality improvement across the European Union, but some degree of policy convergence is also evident
- Having a legal requirement for quality improvement strategies is an important driver of progress, along with the activities of national governments and professional associations and societies
- Patient and service user organisations appear to be having less influence on driving quality improvement
- There is wide variation in the voluntary and mandatory coverage of quality improvement policies and strategies across sectors, potentially leading to varying levels of progress in implementation
- The basic infrastructure for quality improvement is still not present in many healthcare organisations
- The growth of patient mobility across borders, along with the implications of free market provisions for the organisation and funding of healthcare systems in European Union member states, create a need for a degree of cooperation and learning transfer at a policy level

### National and international differences in quality improvement

Our survey suggests that there are substantial variations in the development of healthcare QI across the EU, both internationally and within some member states where policies and strategies are formulated more at a regional than at a national level. These variations make comparisons difficult, but also create an opportunity for international learning and the exchange of experiences and ideas. While there are some countries in which healthcare QI has been a governmental policy concern for up to 20 years or longer, in most it has become a key concern for policy makers particularly in the past 5–10 years. But there are some countries within Europe where the development of healthcare QI is still relatively new, or even embryonic. In broad terms, these findings concur with those of earlier and smaller international surveys in this area.^4^ ^5^

### Legal frameworks for quality improvement

The existence of a statutory legal requirement to implement QI strategies for healthcare systems and organisations was reported as being perhaps the most important incentive for supporting progress in the development of QI initiatives. The implementation and development of quality policies may therefore be at a more advanced stage in member states which have such a legal requirement, and which have had a legal requirement in place for a substantial period of time. The minority of member states who have not yet enacted legislation to require healthcare systems and organisations to put QI policies and strategies in place may wish to consider the experience of those member states which have done so.

### Who drives quality improvement

In this survey, national governments emerged as the key players in developing QI policies, setting quality standards and targets, and providing guidance and support to organisations on implementation. This reflects the major role that national governments play in healthcare funding and provision in member states. However, it was notable that patient and service user organisations were reported as having the least influence on the development of QI policies. It might be argued that the predominant influence of governments and of the health professions through professional associations and societies means it is more difficult for patient and user groups to have their voices heard, and to play their part in shaping QI policies and strategies. This could mean that those policies and strategies, and the QI activities they produce, reflect a professional- and provider-based view of what constitutes high-quality care.

### Variable progress in policy implementation

Although most member states have some form of legal, statutory requirement for QI in healthcare systems and organisations, and most national governments have issued policy documents which set out their policies and strategies, we found that the extent to which specific QI systems or approaches are required or mandated varies considerably, and the existence of the basic infrastructure for QI at a hospital level is rather less established than one might expect. The data suggest wide variation in the voluntary and mandatory coverage of different QI policies and strategies across sectors, potentially leading to varying levels of progress and coverage in implementation. The basic infrastructure for a viable QI programme is still not present in many healthcare organisations.

### Impact

The survey provides some limited evidence of the early impact of national quality policies and strategies, although those achievements were often described by respondents in terms of the development of capacity and capability for QI in healthcare systems, rather than with direct examples or evidence of improved quality. It appears that there are significant barriers to progressing QI, particularly concerned with funding, leadership, and incentives for both organisations and individuals. Overall, the survey results suggest that healthcare QI has made at least some progress in most countries of the EU, and that in some states there are now quite comprehensive and robust quality assessment and improvement mechanisms in place.

### Limitations

Some limitations of our study have already been noted. The small numbers of respondents overall, and their varying levels of knowledge and experience about sometimes complex policies at a national or subnational level, all suggest that caution should be exercised in interpreting these results, and particularly in making any country-level comparisons. However, later phases of the MARQuIS project in which we undertook surveys at the hospital level and conducted a programme of hospital visits afforded some opportunities for verification through triangulation, and broadly served to confirm and support these findings.

## CONCLUSION

Policies on quality improvement in healthcare have largely developed at a national level in EU member states, and have been driven by largely national concerns. Nevertheless, we observed some degree of policy convergence in areas such as the widespread adoption of legal or statutory requirements for healthcare organisations to put quality improvement systems in place, the development of specific mechanisms such as accreditation programmes, and the recent policy priority accorded to patient safety in many member states. However, it seems unlikely that such natural convergence will produce coordinated or integrated quality systems in healthcare at a European level, unless the ends of coordination and integration are more proactively pursued. The growth of cross-border healthcare, and the implications of free market provisions for the organisation and funding of healthcare systems in EU member states, both create a need for some degree of cooperation at a policy level. More practically, it is clear that many EU member states could benefit by learning from the experience gained and progress made in quality improvement policies and strategies by other countries within Europe.
